# Implementing exposure limits for players in contact team sports: review of principles and practices

**DOI:** 10.3389/fspor.2025.1593766

**Published:** 2025-05-01

**Authors:** Colin W. Fuller

**Affiliations:** Colin Fuller Consultancy Ltd., Sutton Bonington, United Kingdom

**Keywords:** injury, risk, prevention, exposure, workplace

## Abstract

Concerns have been raised that professional athletes taking part in contact team sports, such as football and rugby union, are subject to the risk of post-career adverse health conditions. These health concerns include neurodegenerative diseases resulting from head impacts and osteoarthritis in lower limb joints due to wear and tear. There have been suggestions that athletes in contact team sports should be subject to exposure limitations to mitigate these risks. At the present time, little information or guidance is available for athletes and sport governing bodies about how such limitations should be identified and implemented. The criteria used for defining occupational health concerns and the role and nature of occupational exposure limits are discussed. Consideration is given to whether these criteria have been considered and embraced in research studies examining adverse health conditions in professional sport. Recommendations are presented for how future research studies investigating post-career, sport-related, adverse health concerns should be planned and implemented in order to provide the occupational health information required to make evidenced-based decisions about potential health concerns in professional sport.

## Introduction

It is recognised that most occupational activities create some level of risk to workers' health, safety and well-being. The consequences of some risks, such as falls from height, present immediately while others, such as exposure to asbestos and noise, only become apparent many years later. Management of these occupational health hazards is normally based on risk management principles (1), such as:

• identify workplace hazards and situations leading to adverse health concerns,
• quantify the magnitude and nature of adverse health concerns,
• identify causal links between adverse health concerns and workplace conditions,
• develop and implement control measures to reduce the risks of adverse health concerns to acceptable levels.

Although sport and exercise have a positive impact on people's physical and mental health (2), there is a recognition that these activities can, in some circumstances, create health concerns (3). The majority of people taking part in sport and exercise activities do so on a voluntary basis, as amateur athletes. For professional athletes, however, sport is their occupation; consequently, risks to health associated with these activities should be controlled in the same way as they are for other occupations (4, 5). Athletes' injury and ill-health concerns result from a combination of extrinsic risk factors related to the way a sport is conducted and intrinsic risk factors related to participants in the sport (5). The burden of sport-related injury and ill-health concerns is defined by the product of a concern's incidence and its mean severity (6–8). The burden of non-fatal injuries and adverse health concerns in contact, team sports is high; the burden in professional football, for example, has been shown to be three orders of magnitude higher than that reported for occupations generally regarded as high-risk (6).

Control of injury and ill-health risks at work is achieved through seven generic mitigation approaches (1); namely, elimination of hazards, enclosure of hazards, safe systems of work, employee behaviour, use of personal protective equipment, workplace monitoring and employee health surveillance. The higher a mitigation approach sits in this list the greater its effectiveness, as these measures rate high on passive-active scales of implementation (1). The nature of contact team sports, however, means that sport governing bodies generally rely on mitigation measures sitting in the middle to low range of the list. One common measure employed in high-risk settings involves the use of workplace exposure limits (WELs) (1), which aim to keep employees' exposures to hazardous substances and conditions at acceptable levels. Hazards controlled via this approach include chemicals, asbestos fibres, vibration, noise and ionising radiation. Although there are variations in the way that WELs are defined and implemented in different occupations and different countries (9), common principles underpin their application.

A review of evidence relating to claims of neurological disease amongst retired footballers concluded (10): “*there is currently no direct evidence that head injuries in contact sport are associated with transient acute neurological symptoms suggesting brain dysfunction and/or heading footballs leads to permanent brain damage*”. The authors recommended (10), however, that “*doctors should advocate gold standard management of acute head injuries in football”.* If neurological disease or any other adverse health concern were confirmed as a prescribed occupational disease in any professional sport, WELs should be included as part of a gold standard management approach. At the present time, there has been no discussion about how WELs might be applied in professional sport. The aim of this paper is, therefore, to review the principles and practices by which WELs are implemented and to consider how WELs might be applied within professional contact team sports. Although the UK approach for defining and implementing WELs provides the basis for the discussion presented, the UK approach is similar to those adopted in other countries (9, 11).

## Discussion

### Identifying and confirming adverse health concerns

Occupational hazards are defined by their potential to cause injury and adverse health concerns; whether a hazard creates an unacceptable level of risk and leads to injuries or adverse health conditions depends on the occupational setting (1). In the UK, the Industrial Injuries Advisory Council (IIAC), an independent body, guides Government decisions on the prescription of work-related health concerns. The Council's recommendations are based on reviews of the following issues (12), whilst taking account of the fact that the same adverse health concern may also occur within the general population:

• *Temporality*: does exposure to an occupational hazard precede the adverse health concern? It does not follow, however, that exposure to a hazard prior to an adverse health concern confirms the adverse health concern is a consequence of that hazard.
• *Reversibility*: does elimination of a specified hazard reduce the risk of the adverse health concern occurring? If removal of the hazard is followed by a reduction in the risk of the adverse health concern, the possibility of a cause-and-effect association is increased but not confirmed.
• *Dose-response relationship*: does increased exposure to a hazard increase the probability of the adverse health concern occurring?
• *Consistency of research results*: do different study methodologies indicate the same or similar cause-and-effect association? If they do, the probability of the cause-and-effect association being true is increased.
• *Biological credibility*: is a proposed cause-and-effect relationship consistent with current medical knowledge?
• *Consistency of cause-and-effect relationship*: is a proposed cause-and-effect relationship consistent with accepted cause-and-effect relationships?
• *Specificity*: is the adverse health concern associated with more than one hazard?
• *Strength of cause-and-effect association*: is the risk of acquiring the adverse health concern in an occupational setting greater than the risk of the adverse health concern occurring in the general population?

If the prevalence of a health concern is X% in the general population, the prevalence in an exposed population must be at least double this value (≥2*X%) for the concern to be designated as a prescribed occupational disease. This is based on the precept that with a relative risk (RR) value of ≥2 a worker exhibiting the adverse health concern in an exposed population is more likely than not (i.e., >50%) to have sustained the health concern as a result of the workplace conditions. It should be noted that a RR value ≥2 obtained in a research study may not be statistically significant and also that a statistically significant difference between prevalence values in exposed and control groups does not equate to a RR value ≥2. In situations where the RR value falls within the range 1–2 or where there is insufficient information available about the nature and cause of the risk, it may be considered beneficial to implement mitigation measures to reduce the risk of sustaining the adverse health concern. This approach is referred to as the Precautionary Principle. The status of the Precautionary Principle in occupational health is debatable as, although the principle is accepted by some, it is rejected by others (13, 14).

### Relationship between workplace exposures to a hazard and an adverse health concern (the exposure-response relationship)

If an adverse health concern is confirmed in a workplace, the next step is to identify the relationship between the adverse health concern and exposure to a potential causative factor. Although response levels are generally expected to increase as exposure to a hazard increases, there are various types of exposure-response relationship, such as linear-with-no-threshold, linear-with-threshold, logarithmic, hormetic and J-shaped curves (15). Exposure-response relationships are normally confirmed through appropriate context-specific epidemiological studies (16). Identifying relationships at low dose levels of some hazards, such as radiation, can, however, be problematic (17). In addition, some adverse health conditions, such as cancer, have latency periods before they become apparent, which makes it more difficult to confirm and quantify exposure-response relationships. Whichever type of exposure-response relationship applies, it is important to define the relationship correctly (18, 19), as this relationship can influence the format and value of a prescribed WEL.

WELs are set at values intended to provide safe working conditions (1); the optimum objective being that, if workplace exposures remain below the value set, employees would not sustain the adverse health concern. WELs are normally set at values considered to be acceptable for an average, healthy, adult male worker. WEL values encompass the quantity of a hazard and the period of exposure; the quantity reflects the maximum acceptable level of a hazard in the workplace and the time defines the period of exposure over which the quantity value refers. Various types of WEL are used in occupational settings; the type used will be dependent on the hazard, the format in which the hazard is present and the occupational setting. Most WELs are set as 8-hour time-weighted-average (TWA) values based on a working pattern of 5-working-days/week, 40-hours/week and 48-weeks/year and a 40-year working lifetime. For example:

• *Chemicals*: for exposure by inhalation, two exposure limits are prescribed – an 8-hour TWA and a 15-minute TWA short-term exposure limit (STEL). Both values are measured in mg/m^3^ (20).
• *Noise:* two exposure limits are prescribed – an 8-hour TWA and a peak noise value. Both values are measured in decibels (21).
• *Vibration*: two exposure limits are prescribed – an 8-hour TWA for hand/arm vibration and an 8-hour TWA for whole body vibration. Both values are measured in m/s^2^ (22).

An 8-hour TWA value relates to an 8-hour period within a 24-hour period. If employees are exposed for more than 8 h in a 24-hour period, the exposure burden should be normalised to the 8-hour reference period, in order to assess whether employees have exceeded the 8-hour TWA WEL value.

## Workplace monitoring and health surveillance requirements for work-related adverse health concerns

Irrespective of whether WELs are prescribed for a health concern, UK health and safety legislation requires employers to consider workplace monitoring to ensure that working conditions remain within acceptable levels. Health surveillance is required for employees, if the following criteria are met (23):

• An identifiable adverse health concern is associated with normal work activities,
• There is a reasonable likelihood that the adverse health concern occurs as a result of normal work activities,
• Valid techniques are available to detect the adverse health concern,
• Health surveillance is likely to add to the protection of employees.

Routine workplace risk assessments (1) should provide sufficient information to determine whether there is a reasonable likelihood of an adverse health concern occurring during normal work activities.

### Potential adverse health concerns assessed in UK professional sport

Over the past 30 years, there have been few sport-related research studies assessing post-career health concerns among retired UK professional athletes. Compared to the general population, lower limb osteoarthritis (OA) has been shown to be more prevalent among retired professional footballers (24–27) and neurological disease more prevalent among retired professional rugby (28) and football (29–33) players. There have been three formal IIAC reviews for sport-related adverse health concerns. The first review (34) considered neurological disease among boxers and jockeys and OA of the hip and knee joints of footballers. The IIAC concluded that the available evidence was not sufficient to designate these cases as prescribed occupational diseases. The second review considered neurodegenerative disease in professional sportspersons (35). The IIAC concluded that the evidence available was again not sufficient to prescribe this condition as an occupational disease. The third review examined the specific case for OA of the knee joint in professional footballers. In this review (36), the IIAC again concluded that there was insufficient research evidence to conclude that the risk of sustaining knee OA was doubled in the absence of players sustaining a significant accidental knee injury.

Although relationships between players’ match and training exposures and injuries sustained have been examined and modelled for professional rugby (37, 38) and football (39), the studies were not designed to examine potential associations with post-career adverse health conditions. Two studies of retired professional footballers have concluded that there was a possible exposure-response relationship between ball-heading and neurological concerns (31, 32). A study comprising mainly ex-amateur rugby players concluded that there was an exposure-response relationship between chronic traumatic encephalopathy and the length of their playing careers (40); this study used the length of players' careers as a proxy measure for the total number of head impacts players had completed during their careers. A study of ball-heading by amateur footballers indicated there was a relationship between heading a football 20 times during a training session and transient cognitive impairment in the players post-training session (41). None of these studies, on their own, provided sufficient information to confirm exposure related health concerns or to identify appropriate WELs for football or rugby players.

The Rugby Football Union and Premiership Rugby in England jointly proposed a 30 game-involvements (irrespective of playing time in each game-involvement)/season limit for professional players from the start of the 2024/25 season (42). This decision was based on a research study (43) designed to assess the relationship between previous-season match and training exposures and the incidence and burden of injuries sustained in the current season. Results presented from this study, however, demonstrated that there were no associations between previous-season match exposures and current season match injury incidence; current season match injury burden decreased beyond the 30 game-involvements exposure level proposed. In 2021, The Football Association and professional football leagues in England introduced a limit of 10 high-force headers per week during training sessions, to protect players' welfare (44). This heading limit was introduced under the Precautionary Principle, as there was insufficient information available to confirm that imposing a 10 headers/week limit during training would reduce the prevalence of neurological disease in retired footballers.

## A risk-based proposal for establishing WELs in professional sport

Currently, there is insufficient, consistent information available to confirm or reject the existence of adverse health concerns resulting from participation in professional, contact, team sports (10, 34–36). The IIAC (36) in their review of OA in football highlighted that variations in the definition used for a case in different research studies made it difficult to make cross-study comparisons of study results. At the present time, therefore, mitigation actions to address potential sport-related health concerns, such as neurological disease and osteoarthritis, rely on the application of the Precautionary Principle. To remedy this situation, it is important that epidemiological studies are designed and implemented in a way that provides information in a valid and consistent format to enable robust decisions to be made by the responsible bodies for sport-related occupational health concerns. Furthermore, to pre-empt possible confirmation of sport-related health concerns, it is important that consideration be given to the potential use and format of sport-related exposure limits (SREL) so that a consistent approach can be adopted across sports.

When defining SRELs, consideration should be given to their format, as this is most likely to be different from those currently used in other occupations. In this respect, 8-hour TWA and 15-minute TWA exposure values are unlikely to be the appropriate format for most contact team sports, as players may only compete in one game per week for a limited number of weeks each year. Furthermore, games vary in duration across different sports and across different formats of the same sport. Additionally, the majority of professional athletes' exposure is dedicated to training activities, which in many sports will be significantly different to the players' game exposures. One outcome of a risk assessment would be to determine whether the same SREL was likely to be appropriate for male and female players and for different playing positions within the same team sport.

The following points summarise issues that researchers and sport governing bodies should consider before and when undertaking future studies of sport-related adverse health concerns:

• Researchers should be familiar with the review processes used to prescribe occupational diseases (12),
• Researchers should be familiar with review processes used to prescribe WELs (45),
• Risk assessments should be undertaken to consider potential hazards, risk factors and health consequences associated with sport,
• Review available epidemiological information to assess whether adverse health concerns (RR ≥ 2) exist in the sport being examined.
• Consider whether a SREL would be beneficial as a mitigation measure for managing the risk of an adverse health concern in the sport being examined,
• Identify exposure conditions that lead to the adverse health concern in the sport being examined,
• Identify cause-effect and exposure-response relationships that best describe the relationships found and which are consistent with accepted scientific and medical knowledge,
• Identify the most appropriate type(s) of SREL for managing the exposure-response relationships,
• Collect exposure-response data to formulate an appropriate SREL value,
• Define evidence-based SRELs.
• Implement proposed SRELs,

If it proves necessary or beneficial under the Precautionary Principle to define a SREL for a health condition in a sport, it becomes necessary to undertake long-term evaluation studies and ongoing reviews to assess and confirm the veracity of the prescribed SREL. Injury surveillance studies have been implemented in the UK in professional team sports, such as football (46) and rugby (47) for over 25 years. These studies confirm that the incidence of concussion, which may lead to neurological disease, and of lower limb joint injuries, which may lead to OA, are high in both sports. While the requirement for health surveillance in sport has been discussed previously (48), health surveillance becomes more important if adverse health conditions are prescribed and SRELs have been proposed and implemented.

There is ample information available about the short-term effects of injuries sustained in team sports but limited information about whether these injuries lead to post-career adverse health concerns. Of the research studies implemented, few were designed or the results interpreted from an occupational health perspective. It is essential for researchers studying the presence/absence of adverse health concerns in professional contact team sports to be familiar with the criteria used to decide whether workplace health concerns should be prescribed as occupational health diseases (12). Similarly, researchers undertaking research, with the intention of identifying appropriate exposure limits, should be familiar with the requirements for specifying such measures (45).
